# Nrf2 Inhibits GAPDH/Siah1 Axis to Reduce Inflammatory Reactions and Proliferation of Microglia After Simulating Spinal Cord Injury

**DOI:** 10.2174/0115665240280178231218093609

**Published:** 2024-01-10

**Authors:** Chunhe Sha, Feng Pan, Zhiqing Wang, Guohui Liu, Hua Wang, Tianwei Huang, Kai Huang

**Affiliations:** 1 Department of Orthopaedics, Shanghai Jing'an District Zhabei Central Hospital, Shanghai, 200070, China

**Keywords:** Nrf2, GAPDH/Siah1, signaling pathways, spinal cord injury, microglia, inflammatory reactions

## Abstract

**Objective:**

To explore the effect of nuclear factor erythroid 2-related factor 2 (Nrf 2) on microglial inflammatory response and proliferation after spinal cord injury (SCI) through the glyceraldehyde phosphate dehydrogenase (GAPDH) / Seven in absentia homolog 1 (Siah 1) signaling pathway.

**Methods:**

Human microglia HMC3 was induced by lipopolysaccharide (LPS) to establish a SCI cell model. Microglia morphology after LPS stimulation was observed by transmission electron microscope (TEM), and cellular Nrf2, GAPDH/Siah1 pathway expression and cell viability were determined. Subsequently, the Nrf2 overexpression plasmid was transfected into microglia to observe changes in cell viability and GAPDH/Siah1 pathway expression.

**Results:**

Microglia, mostly amoeba-like, were found to have enlarged cell bodies after LPS stimulation, with an increased number of cell branches, highly expressed Nrf2, GAPDH and Siah1, and decreased cell viability (*P*<0.05). Up-regulating Nrf2 inhibited the GAPDH/Siah1 axis, decreased inflammatory responses, and enhanced activity in post-SCI microglia (*P*<0.05).

**Conclusion:**

Up-regulating Nrf2 expression can reverse the inflammatory reaction of microglia after LPS stimulation and enhance their activity by inhibiting the GAPDH/ Siah1 axis.

## 1 INTRODUCTION

Spinal cord injury (SCI), caused by spinal cord or spinal nerve injury, has shown a rising incidence in recent years, especially among young people, which has a great negative impact on patients' normal work and life [1]. SCI can cause sensory, and motor dysfunction, or even the direct loss of motor ability and paralysis in severe cases, depending on injury degree and site [2]. Therefore, timely treatment of SCI is of great significance to ensure patients’ normal activities of daily living. At present, SCI is mainly treated by steroid hormones, neurotrophic drugs, surgery and other schemes clinically. However, all these treatments have unsatisfactory effects, leaving over 60% of patients not completely recovered with a high likelihood of long-term sequelae (sensory disturbance, neurologi-cal dysfunction, *etc*.) [3, 4]. Given the increasing prevalence of SCI, it is of great significance to find a more effective treatment scheme for the disease.

With the increasing attention paid to the application of molecular targeted immune therapy in the medical field, a thorough understanding of the mechanism of various cells and molecules in the human body is considered to be the key to finding new treatment schemes for various diseases [5]. Nuclear factor erythroid 2-related factor 2 (Nrf2) is an essential regulator in the cellular endogenous antioxidant defense system that controls the expression of antioxidant and electrophilic stress genes while being a key transcription factor for maintaining cell metabolism, redox and protein homeostasis [6, 7]. In a number of previous studies, Nrf2 has been proven to be critical in regulating stress-induced organ, tissue and nerve damage [8, 9]. Hence, we believe that Nrf2 may also have important significance in SCI. In another study conducted by our research team, up-regulating Nrf2 was found to be effective in inhibiting the pathological progression of SCI (data not shown), but its mechanism remains to be further confirmed.

To better clarify the relationship between Nrf2 and SCI, we establish an *in vitro* cell model of SCI to observe the influence of Nrf2 on cell biological behavior, so as to assist the clinical understanding of the role played by Nrf2 in SCI and lay the foundation for subsequent research and molecular target therapy of Nrf2.

## 2 MATERIALS AND METHODS

### 2.1 Cell Data

Human microglia HMC3, supplied by Beijing BeNa Culture Collection, were immersed in a 90%EMEM+10%FBS medium that was placed in a 37°C and 5% CO_2_ incubator for culture. Cell subculture was carried out when 70-80% cell growth density was observed, followed by cryopreservation.

### 2.2 Establishment of SCI Cell Model by Lipopolysaccharide (LPS) Inducement

The subcultured microglia were grouped as model and control groups for inoculation into 96-well culture plates at 6×10^3^ cells/mL. After 24 hours of adherent growth, a SCI cell model was established in the model group by adding a 100 ng/mL LPS-supplemented complete medium for 24 hours [10], while the control group was cultured in a normal medium. Cell morphological characteristics of both groups were observed by transmission electron microscopy (TEM).

### 2.3 Nrf2 Overexpression Plasmid Transfection

Obio Technology (Shanghai) Corp., Ltd. designed the Nrf2 overexpression plasmid and its corresponding negative plasmid. Logarithmic-growth-phase cells in the model group were inoculated into the wells of 6 well culture plates (1×10^5^ cells/mL) and transfected with the Nrf2 overexpression plasmid (Nrf2-oe group) and negative plasmid (Nrf2-NC group) when the cell fusion degree reached 75%, respectively, following the LipofectamineTM 3000 kit instructions. The medium was changed every 2 days, and monoclonal cell lines formed 2-3 weeks later were used for subsequent experiments.

### 2.4 Polymerase Chain Reaction (PCR)

We used a TRIzol reagent to isolate total RNA from cells and then reverse transcribed it into cDNA for reaction with the conditions as follows (40 cycles): 90°C/10min (pre-denaturation), 94°C/20s (denaturation), 62°C/30s (annealing), and 72°C/25s (extension). Then, Nrf2, Siah1, Interleukin-1β/4/6/10 (IL-1β/4/6/10), tumor necrosis factor-α (TNF-α), transforming growth factor-β (TGF-β), Superoxide dismutase (SOD) and malondialdehyde (MDA) levels relative to that of β-actin were calculated by 2^-ΔΔCt^. The primer sequences are shown in Table **1**.

### 2.5 Western Blot

The RIPA-lysed cells were centrifuged to obtain supernatant, and the protein concentration was identified by the BCA method. A 20-μg total protein sample was treated with 10% SDS-PAGE, membrane transfer, and immersion in Nrf2, GAPDH and Siah1 protein primary antibodies, followed by sealing with 5% skimmed milk powder and overnight refrigeration (4°C). The membrane was added with HRP-labeled secon-dary antibody for 2 h of room temperature incubation after three TBST washes (10 min/time) the next day. This was followed by membrane development with an ECL luminescence kit and the gel imaging system, and the subsequent gray value calculation of the bands using Image J software (V1.8.0.112, NIH, Madison, WI, USA).

### 2.6 Detection of Cell Viability

Cells were inoculated into 96-well culture plates with 6×10^3^ cells/mL. Each group was provided with 3 multiple wells, into one of which 10 μL CCK-8 solution was placed at 0, 24, and 48 hours of culture. A microplate reader was used to determine the absorbance (OD) value at the 450nm wavelength 4 hours later, and the cell growth curve was drawn. Similarly, cell proliferation in each group was detected according to the CCK-8 assay described above. Additionally, cells were inoculated into 12-well plates (300/well) for 14 days with the medium changed every 3 days. This was followed by 30 min of dyeing with 2% crystal violet at 37°C and the subsequent counting of cloned cells under the optical microscope to calculate the cloning rate.

### 2.7 Immunofluorescence

Microglia were fixed and blocked with 5% BSA. Cells were incubated overnight at 4°C with primary antibodies against Nrf2 (A0674, ABclonal, 1:200), Siah1 (A23247, ABclonal, 1:200), and GAPDH (A19056, ABclonal, 1:1000). After washing, cells were incubated with the appropriate fluorophore-conjugated secondary antibodies (1:200, Santa Cruz, CA, USA) for 1 hour at room temperature. Nuclei were stained with DAPI. The immunofluorescence signals were visualized using a fluorescence microscope (Nikon, Japan). Images were captured at 400x magnification.

### 2.8 Statistical Methods

This study used SPSS24.0 software for statistical analysis and *P*<0.05 as the significance level. All experiments were run in triplicates. The results were described as mean±standard deviation (X̄±s) and compared between groups using variance analysis and LSD intra-group tests.

## 3 RESULTS

### 3.1 Cell Morphology, Viability, and Apoptosis

**Fig. (1):**
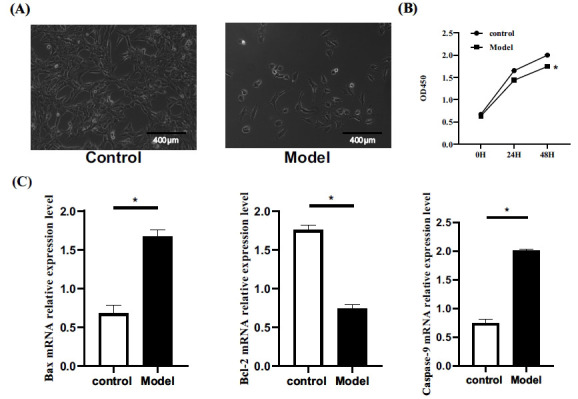
(**A**) Cell morphology. (**B**) Comparison of cell viability. (**C**) Comparison of Bax、Bcl-2 and Caspase-9 mRNA. *P<0.05.

Microscopic analysis unveiled distinct differences: control cells were round or spindle-shaped, while the model group displayed amoeba-like shapes and a significant decrease in cell number (Fig. **1A**). The CCK-8 assay confirmed reduced cell viability in the model group, as evidenced by lower OD values at 24h and 48h compared to the control group (*P*<0.05) (Fig. **1B**). This decline was consistent with elevated Bax and Caspase-9 mRNA expression and decreased Bcl-2 levels in the model group (*P*<0.05) (Fig. **1C**), signifying an augmented tendency for apoptosis.

### 3.2 Comparison of Nrf2 and GAPDH/Siah1 Signal Pathway Expression

**Fig. (2):**
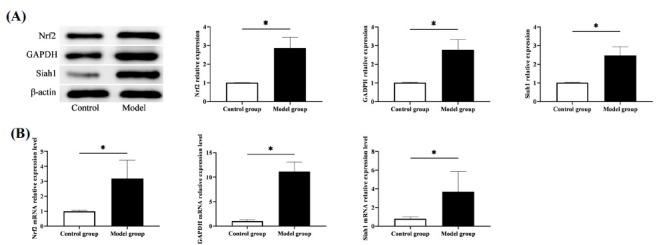
Comparison of Nrf2 and GAPDH/Siah1 signal pathway expression. (**A**) Western blot was performed to detect the expression of Nrf2 and GAPDH/Siah1 signal pathway. (**B**) PCR was performed to detect the expression of Nrf2 and GAPDH/Siah1 signal pathway. *P<0.05.

The PCR quantification revealed higher levels of Nrf2, GAPDH and Siah1 mRNA in the model group versus the control group (*P*<0.05). Similarly, elevated Nrf2, GAPDH and Siah1 protein levels were determined in the model group compared with the control group, as indicated by Western blot analysis (*P*<0.05) (Fig. **2**). Nrf2, GAPDH and Siah1 were upregulated in SCI expression, suggesting their possible involvement in the development of SCI.

### 3.3 Effect of Upregulating Nrf2 on GAPDH/Siah1 Axis in Cells

**Fig. (3):**
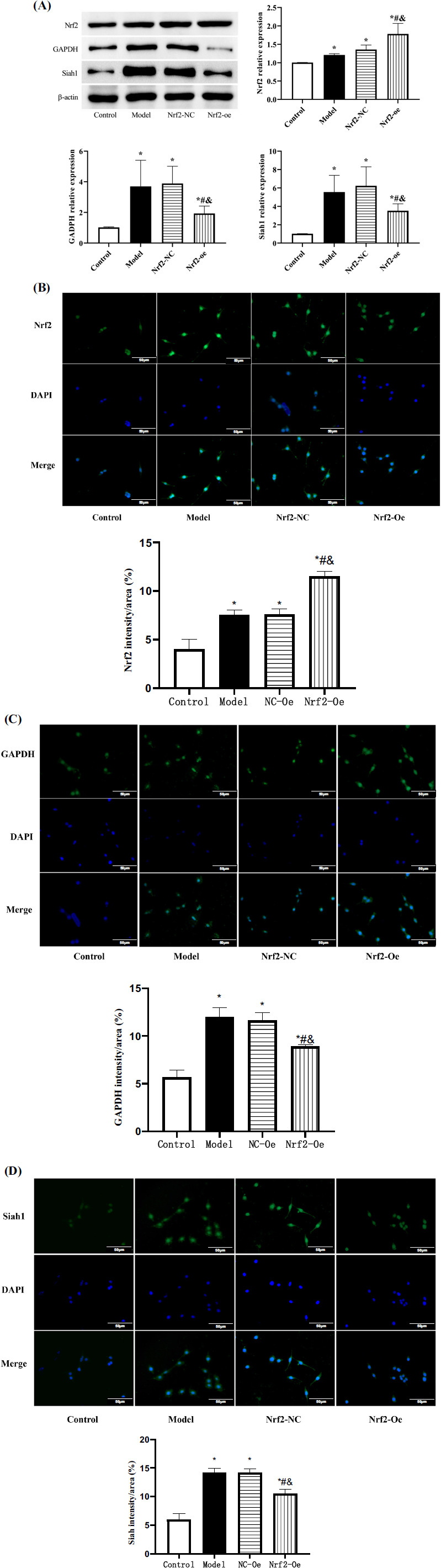
(**A**) Western blot was performed to detect the expression of Nrf2 and GAPDH/Siah1 signal pathway. (**B-D**) Detection of Nrf2 and GAPDH / Siah 1 expression was performed by immunofluorescence. Compared with control group *P<0.05, compared with model group #*P*<0.05, compared with Nrf2-NC group *P<0.05.

After transfection with Nrf2 overexpression plasmid, the Nrf2 expression level was higher in the Nrf2-oe group than in the other three groups (*P*<0.05), while the expression levels of GAPDH and Siah1 were lower than in the model and Nrf2-NC groups and higher than in the control group (*P*<0.05). Model and Nrf2-NC groups showed similar Nrf2, GAPDH and Siah1 levels, higher than those of the control group (*P*<0.05) (Fig. **3**). This suggests that our elevated Nrf2 expression in SCI can inhibit the GAPDH/Siah1 signaling pathway.

### 3.4 Influence of Upregulation of Nrf2 on Cellular Inflammatory Factors and Oxidative Stress

**Fig. (4):**
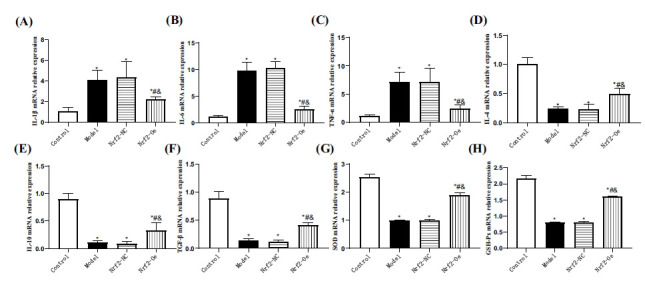
Comparison of the levels of cellular inflammatory factors and OS. (**A**) IL-1β mRNA. (**B**) TNF-α mRNA. (**C**) IL-6 mRNA. (**D**) IL-4 mRNA. (**E**) IL-10 mRNA. (**F**) TGF-β mRNA. (**G**) SOD mRNA. (**H**) GSH-Px mRNA. Compared with control *P<0.05, compared with model #*P*<0.05, compared with Nrf2-NC *P<0.05.

In the model and Nrf2-NC groups, inflammatory factor levels showed no significant differences (*P*>0.05). Pro-inflammatory factors (IL-1β, TNF-α, and IL-6) were highest, while anti-inflammatory factors (TGF-β, IL-10, and IL-4) were lowest among groups (*P*<0.05). Comparatively, the Nrf2-oe group exhibited higher pro-inflammatory and lower anti-inflammatory factors than the control group (*P*<0.05), indicating that Nrf2 elevation in SCI can mitigate pro-inflammatory and enhance anti-inflammatory responses (Fig. **4A**-**4F**). Regarding oxidative stress (OS) assessment, no distinctions were observed in SOD and GSH-Px levels between the model and Nrf2-NC groups (*P*>0.05). However, these levels were lower compared to the control and Nrf2-oe groups (*P*<0.05). Furthermore, SOD and GSH-Px were reduced in the Nrf2-oe group compared to the control group (*P*<0.05) (Fig. **4G**, **H**), suggesting that upregulation of Nrf2 expression can partially counteract OS in SCI cells.

### 3.5 Effect of Up-regulating Nrf2 on Cell Viability

**Fig. (5):**
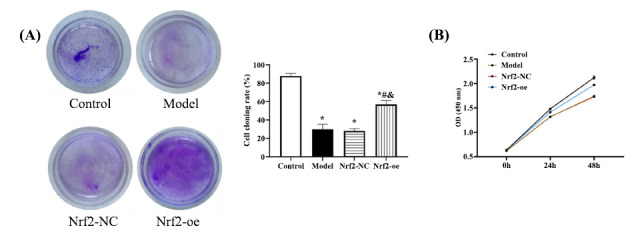
The effect of Nrf2 on cell viability. **A**, Results of cell cloning experiments. **B**, Results of CCK-8 experiments. Compared with control group *P<0.05, compared with model group #*P*<0.05, compared with Nrf2-NC group *P<0.05.

Finally, the observation of cell growth revealed no evident difference between Nrf2-NC and model groups (*P*>0.05), whose cell growth curve and cloning rate were the lowest among the groups (*P*<0.05). While the Nrf2-oe group showed enhanced cell viability that was still lower compared with the control group (*P*<0.05) (Fig. **5**). This result suggests that elevating Nrf2 expression in SCI can promote cellular viability.

## 4 DISCUSSION

SCI can be divided into either primary or secondary injury, of which the former usually results in the destruction of spinal nerve parenchyma and axon network due to trauma, while the latter is a series of pathophysiological cascade reactions based on primary injury [11]. Microglia are widely distributed in the central system and play the role of immunological surveillance. After LPS stimulation, microglia are rapidly activated, resulting in the increased release of iNOS, TNF-α, IL-6 and other substances, which increase inflammation and aggravate injury [12]. Therefore, inducing microglia by LPS is also the most commonly used means to establish an *in vitro* model of SCI in clinical research. After LPS induction, the cell body of cells (mostly amoeba-like) in the model group was enlarged, the number of cell branches increased, and the count decreased, which accords with the pathological manifestations of SCI cells [13], confirming the success of modeling. Up-regulating Nrf2 effectively ameliorated the inflammatory damage of SCI cells, which undoubtedly lays a solid foundation for the future molecular targeted therapy of Nrf2.

In this study, we found that Nrf2 was obviously overexpressed in LPS-induced cell models of SCI, which confirmed its important role in SCI. The high expression of Nrf2 in various stress injury diseases has also been demonstrated in other studies [14, 15], which can also support the accuracy of our results. In the subsequent research, up-regulating Nrf2 led to markedly inhibited levels of inflammatory factors in post-SCI microglia and increased anti-inflammatory factors and cell viability, indicating that up-regulating Nrf2 can inhibit the injury process of SCI in microglia, which is paradoxical with the above-mentioned Nrf2 expression detection results. Nrf2, as we all know, is an important antioxidant transcription factor in eukaryotes and a member of the Cap'n'Collar (CNC)/basic leucine zipper (bZIP) transcription factor family, which is encoded by the erythroid-derived 2-like 2 (NFE2L2) gene and can recognize antioxidant response elements (AREs) [16]. It is characterized by the CNC structure, which contains seven conserved domains, namely Nrf2-ECH homology1-7 (Neh1-7) [17-19], with different functions: Neh1, with a leucine zipper structure (bZip structure), binds to the small Maf proteins in the nucleus and recognizes the ARE sequence, initiating the transcription of antioxidant factors and detoxi-fication genes and playing a vital part in maintaining Nrf2 stability. Neh2 has two binding sites (ETGE motif and DLG motif) that are mainly responsible for binding the negative regulator of Kelch-1ike ECH-associated protein l (Keap1)-Nrf2, and contains many cysteine residues, which is essential in Nrf2 ubiquitination and proteasome-mediated degradation. Neh3 is located at the C-terminal of Nrf2 and is mainly responsible for maintaining the stability and transcriptional activity of the Nrf2 protein. Neh4 and Neh5, located between Neh1 and Neh2, are two independent trans-activation domains, that are mainly involved in the transcription of AREs, the downstream target genes of Nrf2. Neh6 is a serine-rich domain that modulates Keap1-dependent negative regulation. Neh7 inhibits Nrf2 gene transcription after binding to retinoic acid X receptor α. We speculated that the 7 domains of Nrf2 will be activated step by step when SCI occurs. At the initial stage of injury, the activation of Nrf2 will be accelerated because the Neh1-3 domain needs to activate the antioxidant capacity of Nrf2 to maintain its dynamic activity, resulting in a transient high expression state of Nrf2. With the aggravation of damage and the gradual reaction of the Nrf2 domain, Nrf2 will also be consumed by a large number of invading oxidative substances such as reactive oxygen species (ROS) and malondialdehyde (MDA), plus the gradually decreased Nrf2 activity due to the destruction of its original stable structure, this indicates that Nrf2 expression is not sufficient to maintain its stability in spinal cord injury and its function to exert antioxidant effects is reduced. At this time, exogenous reactivation of Nrf2 expression can re-stimulate the antioxidant effect of Nrf2, thus repairing the oxidative stress of organs, tissues and cells. We also obtained results consistent with the above conclusions when testing the OS of each group of cells. Similarly, in some studies, we can also see the low expression of Nrf2 in the late stage of stress injury, and the injury process can also be reversed by activating Nrf2 [20, 21], which undoubtedly proves our point of view. It can be seen that Nrf2 itself presents an elevated state when there is inflammation or injury, but the increase at this time is a feedback-resistance state and an early warning.

In addition, GAPDH and Siah1 in the model group were both increased, which indicates activated GAPDH/Siah1 pathway in SCI, consistent with the results of previous studies [22, 23]. The GAPDH/Siah1 axis is known as an extremely important signal transduction pathway in SCI as well as an activator of stress response [24, 25]. In the research of Tristan CA *et al.* and Su BX *et al*., Nrf2 and GAPDH/Siah1 are considered to play an important role in regulating DNA damage [26, 27]. In our earlier research, significant activation of the GAPDH/Siah1 axis was also observed in SCI rats. However, after up-regulating Nrf2, GAPDH and Siah1 decreased obviously, suggesting that Nrf2 can inhibit the GAPDH/Siah1 pathway, which may be directly related to the above inference, that is, the increasing Nrf2 can effectively resist oxidative damage and maintain the integrity and activity of DNA, thus inhibiting the activation state of GAPDH/Siah1.

In the follow-up research, we also need to carry out clinical trials as soon as possible to confirm the correlation of Nrf2 with SCI, as well as more in-depth and comprehensive experiments on the mechanism and pathway of Nrf2 in SCI, so as to provide more comprehensive reference and guidance for the future molecular targeted therapy of Nrf2.

## CONCLUSION

Nrf2 presents high expression in SCI cells, and up-regulating its expression can effectively reverse inflammatory reactions in microglia after LPS stimulation and enhance cell viability, the mechanism of which is related to inhibiting GAPDH/Siah1 activation. Future molecular therapeutic pathways that target to elevate Nrf2 may provide novel therapeutic options for SCI.

## Figures and Tables

**Table 1:** Sequence of primers.

	**F (5’-3’)**	**R (5’-3’)**
Nrf2	CAATGAGGTTTCTTCGGCTACG	AAGACTGGGCTCTCGATGTG
GAPDH	CAUGUACCAUCAAUAAAGUACCCUG	CAGGGUACUUUAUUGAUGGUACAUG
Siah1	ACCTCGAAGTCCACCATCC	ACTGCATCATCACCCAGTCA
Bax	ATGGACGGGTCCGGGGAGCA	CCCAGTTGAAGTTGCCGTCA
Bcl-2	GTGAACTGGGGGAGGATTGT	GGAGAAATCAAACAGAGGCC
Caspase-9	AGTTCCCGGGTGCTGTCTAT	GCCATGGTCTTTCTGCTCAC
IL-1β	CAACCAACAAGTGATATTCTCC	GATCCACACTCTCCAGCTGCA
IL-4	GGTCTCAACCCCCAGCTAGT	GCCGATGATCTCTCTCAAGTGAT
IL-6	GATGTTGCTGCTTCACTTC	CCTTGTTGGCTTATGTTCTG
IL-10	CTTTAAGGGTTACTTGGGTTGC	CCACTGCCTTGCTTTTATTC
TNF-α	CTCTTCTCATTCCTGCTTG	CTCCACTTGGTGGTTTGCT
TGF-β	AGCTGTACATTGACTTCCGCAAGG	CAGGCAGAAGTTGGCGTGGTAG
SOD	CACAACTGGTTCACCGCTTG	GCCCAACCAGACAGAGAATGA
GSH-Px	CGTGCAATCAGTTCGGACC	CCAGGCATCTCCCTTCCATTC
β-actin	CTAAGGCCAACCGTGAAAAG	ACCAGAGGCATACAGGGACA

## Data Availability

The datas used and/or analyzed during the current study are available from the corresponding author.

## LIST OF ABBREVIATIONS

GAPDH: Glyceraldehyde phosphate dehydrogenase
SCI: Spinal cord injury
Nrf 2: Nuclear factor erythroid 2-related factor 2

## PERMISSION STATEMENT

All images are produced by us, there are no copyright issues involved and can be published in article.
